# Transcriptomic profile of tobacco in response to *Tomato zonate spot orthotospovirus* infection

**DOI:** 10.1186/s12985-017-0821-6

**Published:** 2017-08-14

**Authors:** Changjun Huang, Yupeng Cun, Haiqin Yu, Zhijun Tong, Bingguang Xiao, Zhongbang Song, Bingwu Wang, Yongping Li, Yong Liu

**Affiliations:** Yunnan Academy of Tobacco Agricultural Sciences, Key Laboratory of Tobacco Biotechnological Breeding, National Tobacco Genetic Engineering Research Center, Kunming, 650021 China

**Keywords:** *Tomato zonate spot virus*, Next-generation sequencing, Transcriptome analysis, Plant-virus interaction

## Abstract

**Background:**

*Tomato zonate spot virus* (TZSV), a dominant species of thrips-transmitted orthotospoviruses in Yunnan and Guangxi provinces in China, causes significant loss of yield in lots of crops and is a major threat to incomes of rural families. However, the detailed molecular mechanism of crop disease caused by TZSV remains obscure.

**Methods:**

Next-generation sequencing (NGS)-based transcriptome analysis (RNA-seq) was performed to investigate and compare the gene expression changes in systemic leaves of tobacco upon infection with TZSV and mock-inoculated plants as a control.

**Results:**

De novo assembly and analysis of tobacco transcriptome data by RNA-Seq identified 135,395 unigenes. 2102 differentially expressed genes (DEGs) were obtained in tobacco with TZSV infection, among which 1518 DEGs were induced and 584 were repressed. Gene Ontology enrichment analysis revealed that these DEGs were associated with multiple biological functions, including metabolic process, oxidation-reduction process, photosynthesis process, protein kinase activity. The KEGG pathway analysis of these DEGs indicated that pathogenesis caused by TZSV may affect multiple processes including primary and secondary metabolism, photosynthesis and plant-pathogen interactions.

**Conclusion:**

Our global survey of transcriptional changes in TZSV infected tobacco provides crucial information into the precise molecular mechanisms underlying pathogenesis and symptom development. This is the first report on the relationships in the TZSV-plant interaction using transcriptome analysis. Findings of present study will significantly help enhance our understanding of the complicated mechanisms of plant responses to orthotospoviral infection.

**Electronic supplementary material:**

The online version of this article (doi:10.1186/s12985-017-0821-6) contains supplementary material, which is available to authorized users.

## Background

Orthotospoviruses, the group of plant-infecting members in family *Tospoviridae* (order *Bunyavirales)*, are exclusively transmitted by thrips in nature in the circulative propagative manner [1]. Orthotospoviruses have a broad host range, infecting more than 1000 plant species that include lots of important vegetable, legume, ornamental crops, and weeds. Most orthotospoviruses are characterized by spherical, membrane bound particles (80–120 nm in diameter) containing a tripartite genome of single-strand (ss) RNA, referred to as large (L), medium (M) and small (S) according to their size of genomic segments [2]. In general, the orthotospovirus genome encodes for four structural proteins and two non-structural proteins. The L RNA is in negative sense and encodes the RNA-dependent RNA polymerase (RdRp) on the viral complementary (vc) RNA, and the M RNA is in ambisense polarity and encodes for the precursor of two glycoproteins (Gn and Gc) and a non-structural protein (NSm) on the vc and viral (v) RNA respectively. The S RNA segment is in ambisense polarity and encodes the nucleocapsid (N) protein and another non-structural protein (NSs) on the vc and vRNA respectively. Based upon the N protein sequence, more than two dozen known *Orthotospovirus* species group into at least five distinct phylogenetic clades: *Tomato spotted wilt virus* (TSWV), *Soybean vein necrosis virus* (SVNV), *Iris yellow spot virus* (IYSV), *Watermelon silver mottle virus* (WSMoV), and *Groundnut yellow spot virus* (GYSV) [3]. Furthermore, geographical delineation of distinct clades occurs with origin for each orthotospovirus, such as WSMoV and GYSV clades are classified into Asia group, TSWV and SVNV clades are classified into Americas group and IYSV clade belongs to Europe /Asia group [2, 3].

*Tomato zonate spot virus* (TZSV) was recently isolated in Yunnan province, China as a new orthotospovirus species belonging to WSMoV clade, which corresponds to geographically based original for Asia clade [4]. The results from field investigations and laboratory inoculations indicated that TZSV, similar as TSWV, has a wide host range that includes both agricultural crops and ornamental plant species [4–7]. As one of the most economically important member of both *Orthotospovirus* and WSMoV (Asia) clade, two studies have been conducted to reveal the clustering pattern and cellular distribution characteristics of TZSV in host plant cells and the relationship between TZSV and its vector [8, 9]. However, molecular mechanisms associated with pathogenesis and symptom in the host plant of TZSV remains to be elucidated.

Gene expression profiling analysis, such as microarray represents a well-established technology that has been widely exploited and a vast amount of gene expression data has been accumulated in the last decades, particularly in regard to host-pathogen interactions. Today, next generation sequencing (NGS) technologies including RNA-Seq and digital gene expression (DGE) have created innovative ways to quickly identify a large numbers of genes involved in response to biotic and/or abiotic stress. Due to its ability to provide a deep and precise description of the entire transcriptome, RNA-seq technology has rapidly become a popular tool for genome-wide expression profiling. Up until now, multiple studies have elucidated a nearly complete picture of inducible defense response pathways against various virus infections using this technique [10–18]. Through comparing RNA-seq data from diseased and control plant hosts, sets of genes activated or repressed in varying plant-virus systems have been revealed. However, similar studies by using RNA-seq analysis to illumonate the underlying responsive expression patterns of orthotospovirus infection have not been conducted.

In the present study, transcriptome level of tobacco plants (*Nicotiana tabacum* cv. K326), an important natural host of orthotospoviruses, in responses to TZSV infection was analyzed by using next-generation deep sequencing approach. We investigated the global gene expression changes between virus-infected and mock-inoculated samples. The results indicated that genes involved in photosynthesis and the chlorophyll metabolism pathway were significantly suppressed with TZSV infection. In addition, TZSV infection can potentially perturb primary metabolism pathway of tobacco and activate plant-pathogen interaction, cause changes in endoplasmic reticulum stress and secondary metabolism pathways, such as sesquiterpenoid, triterpenoid, flavonoid, and phenylpropanoid biosynthesis. Our study has provided further insight into the gene expression profiling in orthotospovirus-infected compatible hosts. To best of our knowledge, this is first report of global transcriptome monitoring of host responses to TZSV infection.

## Methods

### Plant growth and virus inoculation

Tobacco plants grown in insect-free growth chamber at a temperature of 23 ± 2 °C and under a light intensity of 200 μmol m^−2^ s^−1^ (14 h photoperiod). TZSV isolate described previously [6] was stored in a freezer at −70 °C. *N. tabacum* plants were mechanically inoculated with the sap of TZSV at one month after planting (four to six-leaf stage). Viral inoculum (the TZSV sap) was prepared from approximately 1 g TZSV infected leaf tissue and homogenized in 10 ml inoculation buffer (0.1 M phosphate buffer, pH 7.0, 0.2% sodium sulfite and 0.01 M 2-mercaptoethanol). Mock-inoculated plants, used as control, were subjected to the same protocol using healthy leaf tissue. ELISA and RT-PCR assays were performed as described previously [6] to confirm virus infection in systemic leaves.

### RNA extraction, RNA-Seq library construction and sequencing

Total RNA was extracted from systemic leaves of TZSV-infected and mock inoculated plants at 12 days post inoculation (dpi) by using TRIzolH Reagent (Invitrogen, San Diego, USA). RNA was treated with DNase I (Invitrogen) to remove residual genome DNA and its quality was verified using a 2100 Bioanalyzer RNA Nanochip (Agilent, Santa Clara, CA).

For RNA-Seq, equal quantities of total RNA from three biological repetitions were mixed and the pooled RNA samples were prepared respectively. Sequencing libraries were constructed by using 3 μg pooled RNA sample as input material and Illumina TruSeq™ RNA Sample Preparation Kit (Illumia, San Diego, USA) following the manufacturer’s recommendations, respectively. For subsequent documentation, a tetrad index code was added to each sample. To obtain high quality mRNA, total RNA sample was purified by using poly-T oligo-attached magnetic beads. High quality mRNA was then used to synthesize first strand cDNA by using random primer and SuperScript II and subsequent second strand cDNA by using DNA polymerase I and RNase H. To select cDNA fragments of preferentially 150–200 bp in size, AMPure XP system (Beckman Coulter, Beverly, USA) was utilized to purify the library fragments. DNA fragments with ligated adaptor molecules on both ends were selectively enriched using Illumina PCR Primer Cocktail in a 10 cycle PCR reaction and then purified using AMPure XP system. The quality of library was assessed on the Agilent Bioanalyzer 2100 system and the clustering of the index-coded sample was performed on a cBot Cluster Generation System using TruSeq PE Cluster Kit v3-cBot-HS (Illumia) according to the vender’s instructions.

Finally, the Hiseq™ 2500 platform (Illumia) was used for the library sequencing to generate 2 × 100 bp paired-end reads and for original image processing of sequences, base calling, and quality value calculations.

### Data filtering, de novo assembly and gene functional annotation

Raw data (raw reads) of fastq format were processed using in-house perl scripts and low quality reads and the adapter sequences from raw data were removed to obtain clean reads. High quality clean data were obtained by calculation of Q score, GC-content and sequence duplication level and subsequently used within all the downstream analyses.

Unigenes were generated by de novo assembly using Trinity [19] and annotated by basic local alignment search tool (BLAST) searching (E-value ≤10^−5^) against seven different databases, including Nt (NCBI nucleotide sequences), Pfam (protein family), Nr (NCBI non-redundant protein sequences), Swiss-Prot (A manually annotated and reviewed protein sequence database), KOG (euKaryotic Ortholog Groups), GO (Gene Ontology) and KO (Kyoto Encyclopedia of Genes and Genomes Ortholog database). Priority order of Nr, Swiss-Prot, KO, and KOG was used to determine the sequence annotation of unigenes if results obtained using different databases conflicted with each other. Coding sequences (CDS) were extracted from unigene sequences and translated into peptide sequences based on information obtained from BLAST. ESTScan was recruited to predict coding regions and determine sequence direction of unigenes if no hits obtained in BLAST analysis.

### Differential gene expression and pathway analysis

The edgeR program package was utilized to adjust the read counts by through one scaling normalized factor [20] and DEGseq R package was used to perform differential expression analysis for each sequenced library [21]. Q-value is a *p*-value that has been adjusted for the False Discovery Rate (FDR) [22] and the threshold for significantly differential expression was set as Q-value < 0.005 and |log_2_ (fold change)| > 2.

The R packages, GOseq, which based Wallenius non-central hyper-geometric distribution and can adjust for gene-length bias in differentially expressed genes (DEGs), was implemented to perform GO enrichment analysis of the DEGs [23]. The metabolic pathway was constructed based on KEGG database and the statistical enrichment of DEGs within constructed metabolic pathways was tested by KOBAS software [24].

### Quantitative RT-PCR (qRT-PCR) analysis

To confirm the results of transcriptome analysis, the expression levels of 28 selected genes were measured in systemic leaves of mock- and TZSV-infected *N. tabacum* using qRT-PCR. Total RNAs were extracted using TRIzol reagent (Invitrogen) and treated with DNase I (TaKaRa, Dalian, China) to remove residual genome DNA. The concentration of total RNA was adjusted to 1 μg/μl with nuclease-free water and first-strand cDNA was synthsized using a PrimeScript First Strand cDNA Synthesis Kit (TaKaRa). qRT-PCR assay was performed on the LightCycler 480^@^ II machine with LightCycler 480^@^ SYBR I Master PCR mix (Roche Applied Science, Basel, Switzerland). Thermocycling conditions were as follows: 95 °C for 5 min; and 40 cycles of 95 °C for 10 s, 60 °C for 15 s, and 72 °C for 20 s, followed by melting curve generation (68 °C to 95 °C). Primers used in qRT-PCR assay were designed using the Premier 6 (PremierBiosoft, Palo Alto, CA) software and shown in Table S1 (Additional file 1: Table S1). To normalize the amount of cDNA in each reaction during qRT-PCR, an internal control gene primers that target the *glyceraldehyde-3-phosphate dehydrogenase* (*GAPDH*) was used with above PCR reagents and conditions. Three biological and three technical replicates were used for each experiment to limit the effect of biological variation and random noise associated with equipment.

## Results

### Illumina sequencing and de novo assembly of sequencing reads

**Fig. 1:**
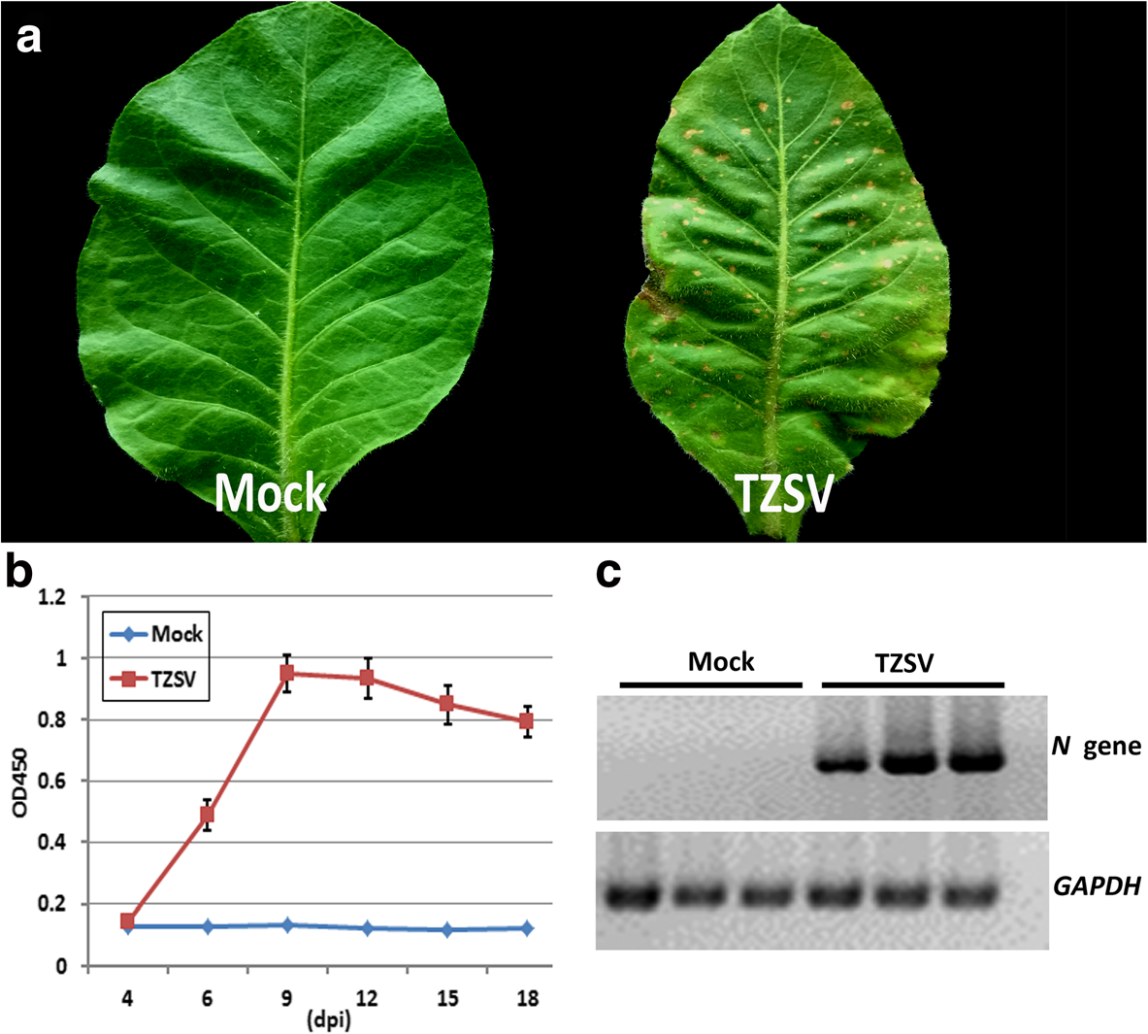
*Tomato zonate spot virus* (TZSV) symptom and detection of virus in systemically infected *Nicotiana tabacum* leaves. **a** Symptoms elicited in *N. tabacum* systemic leaf by TZSV (right) and mock (left) inoculation at 12 days post inoculation (dpi). **b** Time course of TZSV accumulation in *N. tabacum* systemically infected leaves at 4, 6, 9, 12, 15 and 18 dpi. **c** RT-PCR amplification of *N* gene of TZSV from systemically infected leaves of *N. tabacum*

The TZSV inoculated *N. tabacum* leaves developed typical orthotospovirus-like symptoms at 12 dpi. Chlorosis, necrosis spots, and leaf deformation was observed in systemic infected leaf tissues (Fig. 1a). To determine the best time points to analyze transcriptional responses to systemic TZSV infection, viral accumulation was analyzed through an ELISA time-course experiment. The results of absorbance values obtained from the ELISA reaction indicated the levels of TZSV rise from 4 to 9 dpi and then descend slowly for the next 9 days (Fig. 1b). In our attempt to reveal molecular mechanisms underlying pathogenesis and symptom development, symptomatic systemic leaves were collected at 12 dpi for RNA extractions from TZSV infected and mock-inoculated plants. To reduce biological errors caused by natural variation, three TZSV inoculated plants that exhibited similar symptoms and three mock inoculation plants were harvested to prepare one pooled RNA sample respectively. RT-PCR analysis of the six total RNA samples was performed to validate that symptomatic leaves were infected by TZSV (Fig. 1c).

Two cDNA libraries, TZSV_12 (TZSV inoculated leaf material) and Mock_12 (mock-inoculated control), were constructed and used to perform Illumina deep sequencing. After filtering raw data by removing adaptors sequence and reads of unknown or low-quality nucleotides, we obtained 47.3 and 55 million clean reads that encompassed 5.91 Gb and 6.88 Gb of sequence data for mock- and TZSV-infected plants respectively (Table 1). After de novo assembly using the Trinity program, 135,395 unigenes were obtained from TZSV_12 and Mock_12 combined libraries with lengths from 200 to 17,637 bp (Additional file 2: Figure S1) for further analysis.

**Table 1:** Summary statistics for sequencing for two libraries from mock- and TZSV-infected *N. tabacum* plants

	Mock_12	TZSV_12
Raw Reads (bp)	55,159,660	47,420,120
Clean reads	55,017,810	47,300,730
Clean bases	6.88 Gb	5.91 Gb
Q20 (%)	95.52	95.55
Q30(%)	91.35	91.42
GC(%)	43.81	42.94

### Annotation of unigenes in tobacco

To annotate the assembled unigenes, a BLAST search was conducted against seven public databases (Nr, Nt, KO, Pfam, Swiss-Prot, KOG, KEGG and GO). The results indicated that 102,771 (75.9%) unigenes were matched to one or more of the databases, and 73,095 (53.98%), 96,607 (71.35%), 23,859 (17.62%), 48,241 (35.62%), 49,041(36.22%), 49,179 (36.32%), and 16,880 (12.46%) of which were matched to Nr, Nt, KO, Pfam, Swiss-Prot, GO, and KOG respectively (Table 2).

**Table 2:** Summary statistics for sequence assembly and annotation of unigenes

	Number of Genes	Percentage (%)
Total Unigenes	135,395	100
Annotated in NR	73,095	53.98
Annotated in NT	96,607	71.35
Annotated in KO	23,859	17.62
Annotated in SwissProt	48,241	35.62
Annotated in PFAM	49,041	36.22
Annotated in GO	49,179	36.32
Annotated in KOG	16,880	12.46
Annotated in all Databases	10,285	7.59
Annotated in at least one Database	102,771	75.9

### Global gene response to TZSV infection in *N. tabacum*

**Fig. 2:**
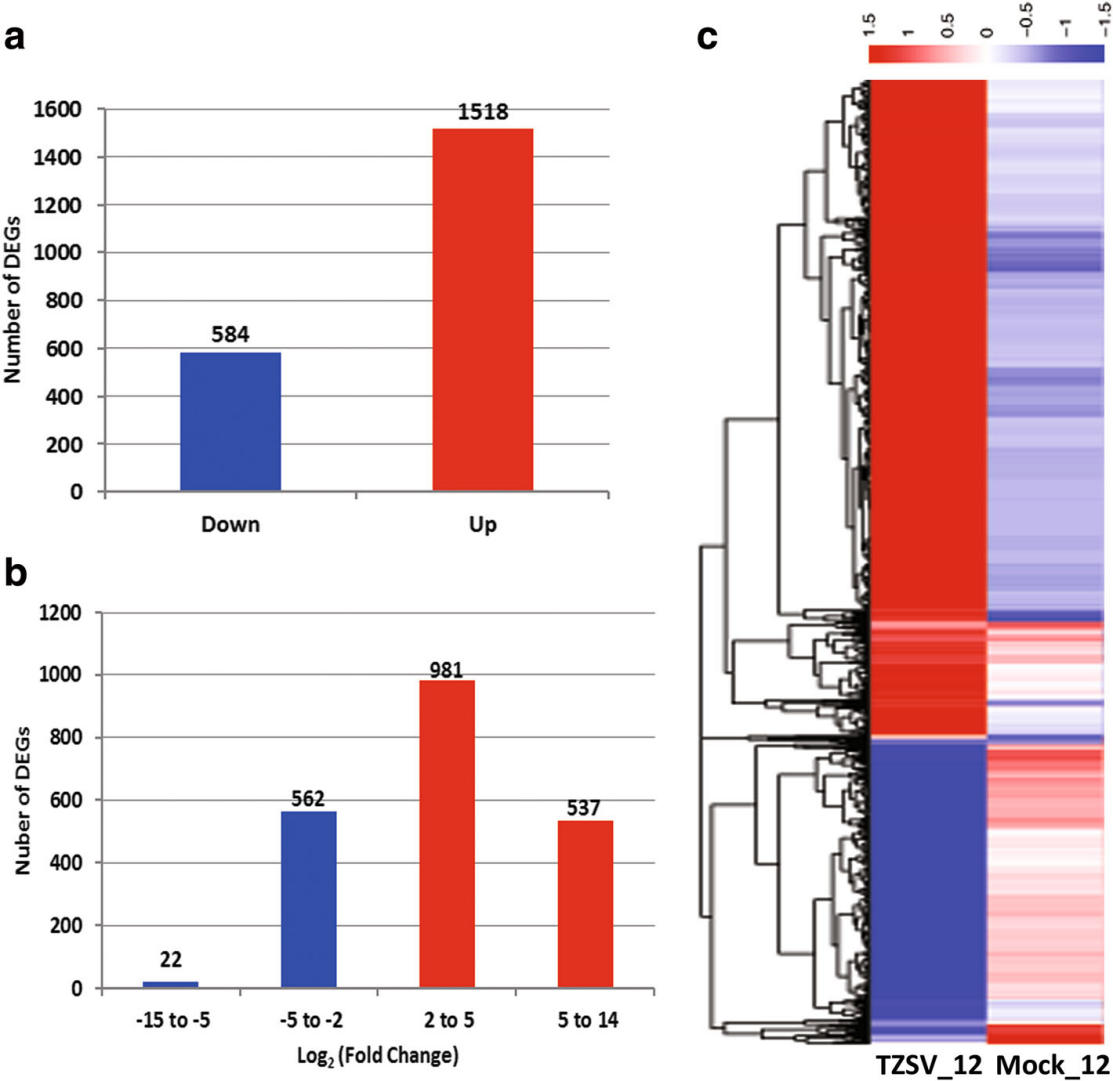
Summary of differentially expressed genes (DEGs) in response to TZSV infection. **a** The number of up-regulated and down-regulated genes in the TZSV infected *N. tabacum* at 12dpi. **b** Value of log_2_ (fold change) distribution of DEGs. **c** Hierarchical clustering of differential expression profiles for 2102 genes between mock- and TZSV -infected plant libraries was based on cut off value q < 0.005 and absolute value of the log_2_ (fold change) >2. Blue represents lower expression, red represents high expression, the rows represent transcriptional units

To identify genes showing a significant expression change in *N. tabacum* upon TZSV infection, DEGseq [20] was used with significance criteria of [Q-value < 0.005&|log_2_(fold change)| > 2]. A total of 2102 DEGs were obtained between the TZSV_12 and Mock_12 samples, among which 584 DEGs were down-regulated and 1518 up-regulated respectively (Fig. 2a). The detected changes [log_2_(fold change)] of DEGs ranged from −15 to +14, and more than 73% of the DEGs were up- or down-regulated between 2 to 5 fold (Fig. 2b). Changes in the abundances of transcripts between the two groups of mock- and TZSV- infected plants are represented by hierarchical clustering (Fig. 2c).

### Functional classification of DEGs

**Fig. 3:**
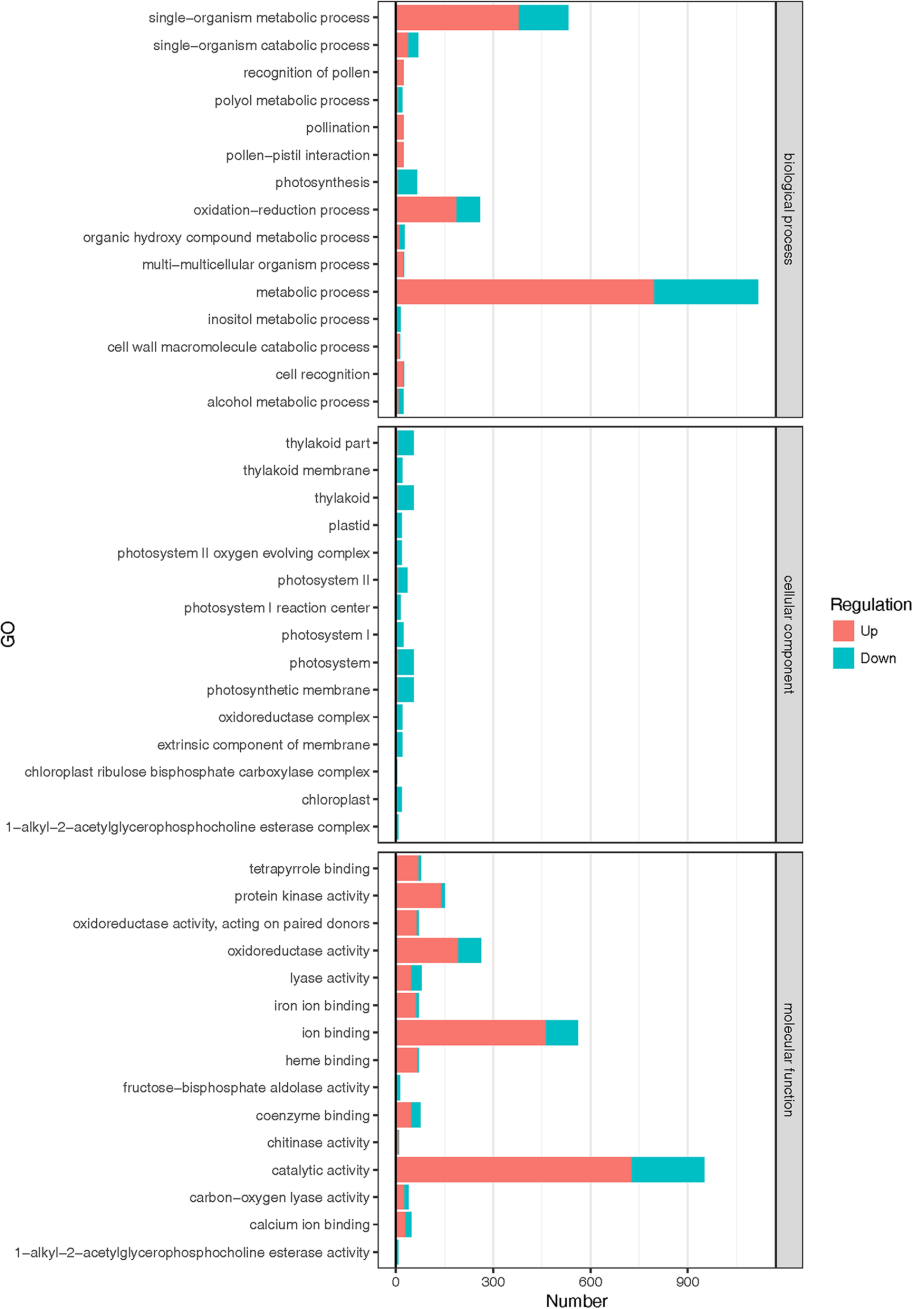
GO Classification of DEGs. 2102 DEGs were divided into three categories and 47 functional sub-groups. The y-axis shows the functional groups and the x-axis shows the number of genes calculated in our library

GO enrichment analysis was conducted on all DEGs to further elucidate the gene functions. Based on sequence homologies, 1642 DEGs were annotated to the GO database and separated into three main categories, biological processes (BP), cellular components (CC) and molecular functions (MF) (Additional file 3: Table S2). Of these, 22 cellular component, 68 molecular function, and 153 biological process were significant (Corrected *P* < 0.05) (Additional file 4: Table S3). For the BP category, response to metabolic process (GO:0008152) comprised the largest proportion of genes in the enriched pathways, followed by single-organism metabolic process (GO:0044710), oxidation-reduction process (GO:0055114), and photosynthesis (GO:0015979) (Fig. 3). Within the CC category, most of DEGs were putatively involved in photosystem and thylakoid development. It is worth noting that almost all genes in the significantly associated with CC category are repressed (Fig. 3). The most enriched groups in the MF category were catalytic activity (GO:0003824), ion binding (GO:0043167), oxidoreductase activity (GO:0016491) and protein kinase activity (GO:0004672) (Fig. 3).

**Fig. 4:**
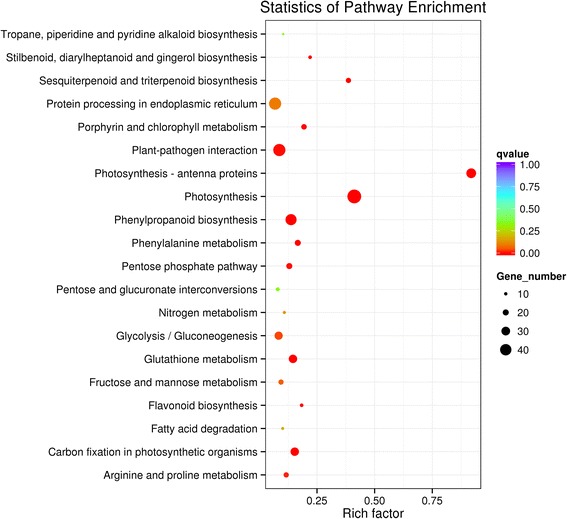
Kyoto Encyclopedia of Genes and Genomes (KEGG) pathway enrichment analyses. The 20 top enriched Kyoto Encyclopedia of Genes and Genomes (KEGG) pathways based on DEGs. The x-axis shows the rich factor. The y-axis shows the pathway names. The size of each point represents the number of genes enriched in a particular pathway. The bigger the value of rich factor and the smaller the value of Q-value indicate the degree of enrichment is more significant

To extent our understanding of molecular and biological functions of the DEGs, all differentially expressed unigenes were mapped to the KEGG database categories and compared the results with the whole transcriptome background. Pathway enrichment analysis identified 93 pathways, of which 14 KEGG pathways were significantly enriched (Corrected *P*-value <0.05) with TZSV infection (Table 3, Fig. 4, Additional file 5: Table S4). Particularly, pathways with functional classes related to processes involving photosynthesis, plant-pathogen interaction, and metabolism were perturbed by virus infection were indicated by the KEGG analysis using all DEGs (Fig 4). To identify more pathways that are really pertinent to phenotypic difference, all significant up- and down-regulated genes were collected and submitted to further pathway enrichment analysis separately. Our results showed that most of unigenes involved in photosynthesis-antenna protein synthesis (ko00196), photosynthesis (ko00195), carbon fixation in photosynthetic organisms (ko00710), and porphyrin and chlorophyll metabolism (ko00860) were down-regulated (Table 3), while genes involved in sesquiterpenoid and triterpenoid biosynthesis (ko00909), phenylpropanoid biosynthesis (ko00940), glutathione metabolism (ko00480), stilbenoid, diarylheptanoid and gingerol biosynthesis (ko00945), plant-pathogen interaction (ko04626), flavonoid biosynthesis (ko00941), arginine and proline metabolism (ko00330), and protein processing in endoplasmic reticulum (ko04141) were up-regulated (Table 3).

**Table 3:** Significantly enriched (*P* < 0.05) KEGG pathways of differentially expressed genes by TZSV infection

Pathway	Pathway ID	*P*-Value	DEGs	
			Up	Down
Photosynthesis - antenna proteins	ko00196	5.41E-27	-	34
Photosynthesis	ko00195	2.12E-26	1	48
Sesquiterpenoid and triterpenoid biosynthesis	ko00909	1.08E-09	17	-
Phenylpropanoid biosynthesis	ko00940	2.87E-08	36	3
Carbon fixation in photosynthetic organisms	ko00710	2.22E-07	6	23
Glutathione metabolism	ko00480	5.77E-07	26	3
Porphyrin and chlorophyll metabolism	ko00860	2.60E-06	2	16
Phenylalanine metabolism	ko00360	5.53E-06	19	1
Stilbenoid, diarylheptanoid and gingerol biosynthesis	ko00945	8.47E-05	11	-
Pentose phosphate pathway	ko00030	0.000133	7	13
Plant-pathogen interaction	ko04626	0.000279	43	-
Flavonoid biosynthesis	ko00941	0.000343	11	-
Arginine and proline metabolism	ko00330	0.001251	17	-
Glycolysis / Gluconeogenesis	ko00010	0.004729	11	17
Fructose and mannose metabolism	ko00051	0.009188	4	13
Protein processing in endoplasmic reticulum	ko04141	0.016025	42	1
Nitrogen metabolism	ko00910	0.024104	5	4
Fatty acid degradation	ko00071	0.034445	9	-

### Validation of expression patterns by qRT-PCR

**Fig. 5:**
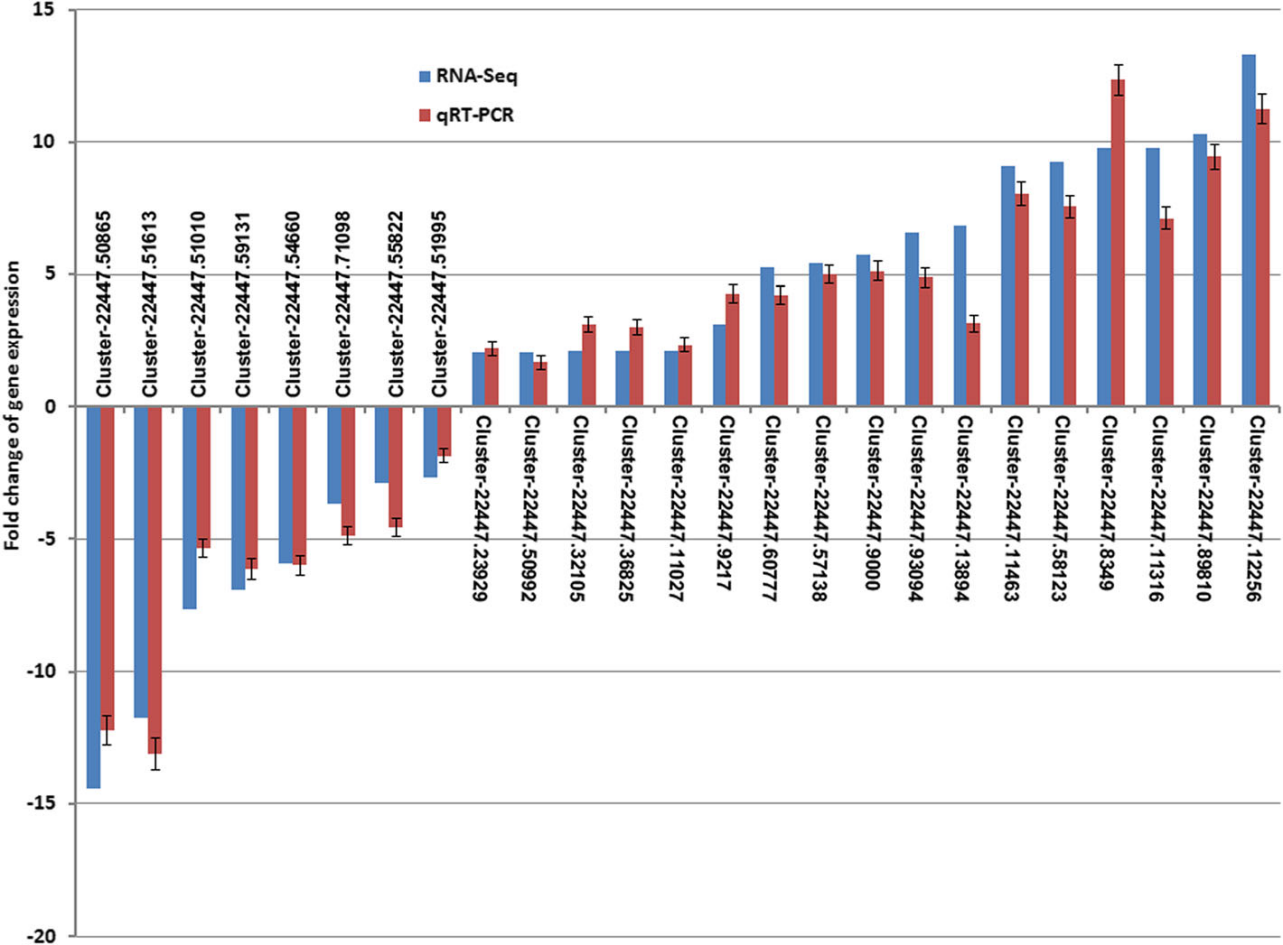
Validation of the expression of selected genes from RNA-Seq using qRT-PCR. Gene IDs of selected genes are positioned above or below the x-axis. The expression of each gene was normalized to endogenous *glyceraldehyde-3-phosphate dehydrogenase* (*GAPDH*) gene

RNA-Seq revealed the expression profiles of thousands of genes. In order to validate the DGE result, qRT-PCR analysis were performed using specific primers of 28 selected unigenes with annotations (Additional file 1: Table S1). The results showed 25 genes were differentially expressed in both DGE and qRT-PCR with a concordant direction of fold change (Fig. 5). Inconsistencies among the remaining 3 genes between qRT-PCR and RNA-Seq could be artificially caused by lack of specific primers targeting regions with high discriminatory or possibly difference in sensitivity of two methods [19]. Nevertheless, the results of qRT-PCR showed an almost similar pattern with those obtained from DGE data, indicating reliable results.

## Discussion

Orthotospoviruses, a highly destructive pathogen of various crops, threatens crop producing areas worldwide [2, 3, 25]. Two previous studies investigated tomato and chrysanthemum host responses to TSWV and the mechanisms underlying disease development using microarrays to monitor transcriptional changes [26, 27]. However, study on gene expression profiles of the Asia clade orthotospovirus such as TZSV is limited. The present work focuses on transcriptional changes in *N. tabacum* leaf in response to TZSV infection as measured using NGS technology. 135,395 unigenes were obtained from RNA-Seq and de novo assembly, of which 2102 genes were significantly differentially expressed in response of TZSV infection (Fig 2). GO and KEGG enrichment analysis were subsequently performed to classify functions of DEGs. Based on the GO classification, these DEGs were assigned to three main categories (BP, CC, and MF), which together contain 243 subcategories (Additional file 3: Table S2) that provide a good indication of the diversity of genes affected by viral infection. Similarly, KEGG annotation showed that these DEGs were distributed in 14 KEGG pathways which also provide insight into the various biological pathways associated with viral infection (Table 3).

Photosynthesis process and chloroplast development were the most significantly changed by TZSV infection. Genes in photosynthesis-antenna protein synthesis (ko00196), photosynthesis (ko00195), porphyrin and chlorophyll metabolism (ko00860), carbon fixation in photosynthetic organisms (ko00710) pathways were globally repressed (Table 3). This result is consistent with leaf chlorosis, which is major viral symptom of TZSV. Chloroplast is not only the main metabolic energy originator, but also the abiotic/biotic stress sensor and defense signal generator [28, 29]. Chlorotic area, where it seems to interfere with chloroplast structure, function and/or development, is usually associated with virus large accumulation and clustering [28–30]. An increasing number of reports on plant-virus combinations have revealed that down-regulation of photosynthesis and chloroplast-related genes are correlated with the development of infection symptoms [12–16]. In addition, recent several evidences have revealed that viral effectors affect chloroplast ultrastructure and symptom through directly interact with chloroplast component [31–36]. Chloroplast’s role in the process of the replication and movement of plant virus and plant defense against virus also been documented [30, 37, 38]. Although the mechanism of viruses how to down-regulate chloroplast- and photosynthesis-related genes is unknown, our study implies that important role of chloroplasts and photosystem in the TZSV infection and pathogenesis.

Abundant metabolism pathways, both primary and secondary metabolism, are identified in response to TZSV infection (Table 3). The majority genes of primary metabolism are repressed and most genes in secondary metabolism are induced. Similar metabolic pattern is found in several metabolism pathways with different plant-virus combinations. Flavonoid biosynthesis (ko00941), phenylalanine metabolism (ko00360), arginine and proline metabolism (ko00330) and glutathione metabolism (ko00480) are enriched in different host plant challenging with diverse viruses, which means these pathways play a central role in plant responses to virus infection [11, 16, 39–42]. One of the most interesting discoveries in our study, which have not been extensively reported in plant-pathogen interaction before, is the mapping of 17 sesquiterpenoid and triterpenoid biosynthesis (ko00909) genes involved in TZSV infection, which are exclusively categorized into solavetivone synthesis pathway (Additional file 5: Table S4). Solavetivone, a low molecular weight phytoalexin, have provided limited proofs of their implication in plant/microorganism interactions [43]. However, Ito et al. reported that solavetivone was particularly detected in *Tobacco mosaic virus* (TMV)-infected *N. tabacum* cv. Samsun NN which bears the resistance *N* gene can produce necrotic local lesions due to hypersensitive reaction (HR) [44]. The regulation of genes involved in solavetivone pathway has important implications for solavetivone is associated with necrosis symptom induced by TZSV. Further studies on the solavetivone profiles of TZSV-infected plants will clarify the role of this phytoalexin.

The endoplasmic reticulum (ER), the largest intracellular organelle, forms a complex network of continuous sheets and tubules which are frequently perturbed and utilized by viruses to create membranous replication factories (RFs) and drive their movement on the ER [45]. Previous study showed that TZSV majorly distribute in the swollen membrane of ER as single particle, which implies that the ER may play pivotal roles during orthotospoviruses infection [9]. Emerging evidences on TSWV and *Groundnut bud necrosis virus* (GBNV) suggest that ER membrane transport system serves as an important direct route for orthotospovirus trafficking [46–48]. In our present study, some of the key genes of ER-associated protein folding such as lumenal binding proteins (BiPs), ER-localized HEAT SHOCK 70 PROTEIN (HSP70) family mumbers, HSP90 family member, HSP40 (DnaJ) family member are up-regulated (Additional file 5: Table S4). ER stress elicits the unfolded protein response (UPR) when plants subjected to severe or chronic biotic/abiotic stress, which promotes programmed cell death (PCD) [49]. Therefore, novel insights into the signaling mechanisms and regulatory networks of the ER stress responses to orthotospovirus infection in plants were provided in our present result.

In long-term interactions with pathogens, a series of defense mechanisms,including HR production, accumulation of pathogenesis-related proteins and enzyme changes (such as peroxidase and polyphenol oxidase) were established in plants [50]. Among the DEGs of TZSV infection, 43 unigenes were annotated to regulate the pathway of plant-pathogen interaction (Table 3). Genes in generation of calcium signals (*CNGC*, *CaM*/*CMLs*) and oxidative burst (*CDPK*, *Rboh*) cascades, which associated with signaling events in pathogen-associated molecular pattern (PAMP)-triggered immunity (PTI), were up-regulated after TZSV infection. Genes involved in in the process of effector-triggered immunity (ETI), like as disease resistance genes (*HSP90*, *RIN4*, *CERK1, EIX1/2*), were also up-regulated in the current condition. In addition, abundant expression of defense-related genes (*WRKYs*, *PR1*) involved in both PTI and ETI processes were observed (Additional file 5: Table S4). The up-regulation a set of transcripts encoding plant disease resistance proteins suggested that the defense system of *N. tabacum* was particularly activated by TZSV infection.

## Conclusions

This is the first transcriptome-wide study of the TZSV-host interaction. High-throughput RNA-Seq analysis was used in this study permitted us to draw some general associations between TZSV infection and global gene-expression changes in the TZSV-infected tobacco. GO and KEGG pathway enrichment analysis revealed biological pathway that are affected in the host plant infected by TZSV, and showed how mock- and TZSV-inoculation trigger a different cascade of molecular changes. Our analyses further our understanding of system level changes associated with TZSV infection, and provide a useful basis for future explorations of symptom development and pathogenesis of TZSV infection.

## Additional files

Additional file 1: Table S1. Primers used for the validation of DEGs. (DOCX 17 kb)

Additional file 2: Figure S1. The distribution of unigene size. (PNG 51 kb)

Additional file 3: Table S2. GO enrichment Result. (XLSX 416 kb)

Additional file 4: Table S3. Significantly enriched GO terms. (XLSX 80 kb)

Additional file 5: Table S4. KEGG pathway enrichment result. (XLS 35 kb)

## Abbreviations

BiPs: Binding proteins
BLAST: Basic local alignment search tool
BP: Biological processes
CC: Cellular components
CDs: Coding sequences
DEGs: Differentially expressed genes
DGE: Digital gene expression
dpi: Days post inoculation
ER: Endoplasmic reticulum
ETI: Effector-triggered immunity
GAPDH: Glyceraldehyde-3-phosphate dehydrogenase
GBNV: Groundnut bud necrosis virus
GO: Gene Ontology
GYSV: Groundnut yellow spot virus
HR: Hypersensitive reaction
HSP70: HEAT SHOCK 70 PROTEIN
IYSV: Iris yellow spot virus
KO: Kyoto Encyclopedia of Genes and Genomes Ortholog database
KOG: euKaryotic Ortholog Groups
MF: Molecular functions
NGS: Next generation sequencing
Nr: NCBI non-redundant protein sequences
Nt: NCBI nucleotide sequences
PAMP: Pathogen-associated molecular pattern
PCD: Programmed cell death
Pfam: Protein family
PTI: PAMP-triggered immunity
qRT-PCR: Quantitative RT-PCR
RdRp: RNA-dependent RNA polymerase
RFs: Replication factories
ss: Single-strand
SVNV: Soybean vein necrosis virus
Swiss-Prot: A manually annotated and reviewed protein sequence database
TSWV: Tomato spotted wilt virus
TZSV: Tomato zonate spot virus
UPR: Unfolded protein response
WSMoV: Watermelon silver mottle virus
