# Supplementary material for: Transcriptomic profile of tobacco in response to Tomato zonate spot orthotospovirus infection
Source: Virol J. 2017 Aug 14;14:153. doi: 10.1186/s12985-017-0821-6 (PMC5557316; doi:10.1186/s12985-017-0821-6)
Supplement: Supplementary file 1 — Primers used for the validation of DEGs. (DOCX 17 kb) [file 12985_2017_821_MOESM1_ESM.docx]

Primers used for the validation of DEGs.

| ID | Sequence 5'-3' |
| --- | --- |
| Cluster-22447.55822F | TGGCAAGAAGAAGCTGACATAGAAGT |
| Cluster-22447.55822R | CCAAGAAGAGAAGGATGTAGCAGAAGA |
| Cluster-22447.59131F | TACATCCGAGCCAATAGCCATCCT |
| Cluster-22447.59131R | AGCAACACTCTTCCCATTTCTCATCTT |
| Cluster-22447.51995F | GTCATTAGCCAGTTGCCTCAGTCTA |
| Cluster-22447.51995R | TGCTGCTACCAGTATCATCAGAAGAAT |
| Cluster-22447.8349F | CCATATCAACATCCTTAGGTAGCATCC |
| Cluster-22447.8349R | TCCAGCAGATACAAGAGTCCTTATCAA |
| Cluster-22447.23929F | TGGCGGAGGCTCAATGGAAGA |
| Cluster-22447.23929R | ACCTGCTCTGTCATCTGTTGGAAC |
| Cluster-22447.11027F | GGAGAGGCGTCAGAGTGAGGAA |
| Cluster-22447.11027R | CAGAATCCAGGCATTGTGTATCTTGTC |
| Cluster-22447.89810F | GCGATGGATGAGATTGAACGGTAAT |
| Cluster-22447.89810R | TCTTGAGTTGACTTGACTTGACACA |
| Cluster-22447.51010F | CTTAGGCGGTGCGGTTGCTT |
| Cluster-22447.51010R | ACGAAAGTGGACATACAAGAAGAAGAC |
| Cluster-22447.11948F | TCCTGCTCCAGAACTTCTTGTAATACT |
| Cluster-22447.11948R | GCCGTCAAGCCTCAACCAGAT |
| Cluster-22447.11463F | TGCTGTAGTGAAGTTGTGTCAATGTCT |
| Cluster-22447.11463R | ACCATCATGCCCAGTCAAGAAGAAG |
| Cluster-22447.51236F | TCGTGTTTAGTTGCCATCGTTGAGT |
| Cluster-22447.51236R | ATAGTGTGACGGGCGGTGTGTA |
| Cluster-22447.9217F | CGGCGACGAGAATAACGATTGATGA |
| Cluster-22447.9217R | GACTGTTCCACCACCGTCCTCT |
| Cluster-22447.54660F | GGTTCTTCCCAGAATTTGTACCACTTT |
| Cluster-22447.54660R | TTCTCCTTGACTTGCGGGTTCTTT |
| Cluster-22447.13894F | CTGGAGGTGTAATGACTGTGTTGATTC |
| Cluster-22447.13894R | CGTTGGTGATGGTTATCTTGTTCTTCT |
| Cluster-22447.60777F | TTGAAGAAGAAGATGACGCCGAAGT |
| Cluster-22447.60777R | GGAACTAGCAAGCCTACTCACTATCA |
| Cluster-22447.12256F | GCAGATGTAGGCGTGGAACCATT |
| Cluster-22447.12256R | GCACATCCAACACGAACCGAGTTA |
| Cluster-22447.36825F | GCTGATCCTGTCCGCACTTACC |
| Cluster-22447.36825R | TAACCACCACCATCGCCATTACTTC |
| Cluster-22447.71098F | TGTTCTCTGAAGTAAGCAAGCACAAG |
| Cluster-22447.71098R | CTGACCAAGTGACCGCTCCATT |
| Cluster-22447.50865F | CATGGATAATGAAGTGGGATGTAGAGT |
| Cluster-22447.50865R | TGTCTGGTGACTTTCTGGAATGGA |
| Cluster-22447.9000F | TTCAAGTCTTGGTCGGGTTTGGATTT |
| Cluster-22447.9000R | TCCTCGCCACGGCCTCAATT |
| Cluster-22447.57138F | ACCTCTTGCTGGCTTATGGGATACA |
| Cluster-22447.57138R | TGGCTTCATCAGTCACGGAGTCAT |
| Cluster-22447.50992F | GTGAGGATTAAGTGCTATGAGGAATGC |
| Cluster-22447.50992R | ATGGTGCTTGCGGTGGATTAGAA |
| Cluster-22447.32105F | TCCTGTCTTCATCATTGCTACGAACTC |
| Cluster-22447.32105R | GCTGATGGATGTGGCTGATGTTGA |
| Cluster-22447.55150F | TTTGACTCCACCTTTGCCTTTAGATTC |
| Cluster-22447.55150R | ACATTGAAGATGACCATTCTCCATTGC |
| Cluster-22447.11316F | AGCCTTATGGGAGTGGTTTCATCAGA |
| Cluster-22447.11316R | GCGTTGTACTTGCTTCTTCACATTGC |
| Cluster-22447.58123F | GAAGACATTGGAGCCTCACGGTTAA |
| Cluster-22447.58123R | TCCTTGTGACGGTCGGCATCT |
| Cluster-22447.51613F | CCGAACGCAGCATACAACTCTCC |
| Cluster-22447.51613R | GAGCACCTAACAACGCATCTTCACA |
| Cluster-22447.93094F | CAGCAACAATTCCTACCACAGCATT |
| Cluster-22447.93094R | AACACCTCTCCAAGAACGACTCAAC |
| GAPDHF | CTGCTATCAAGGAGGAGTCTGAAGGA |
| GAPDHR | ACCACGCTAGGAACTGCCACTT |
