# Supplementary figures and images for: Transcriptomic profile of tobacco in response to Tomato zonate spot orthotospovirus infection
Source: Virol J. 2017 Aug 14;14:153. doi: 10.1186/s12985-017-0821-6 (PMC5557316; doi:10.1186/s12985-017-0821-6)

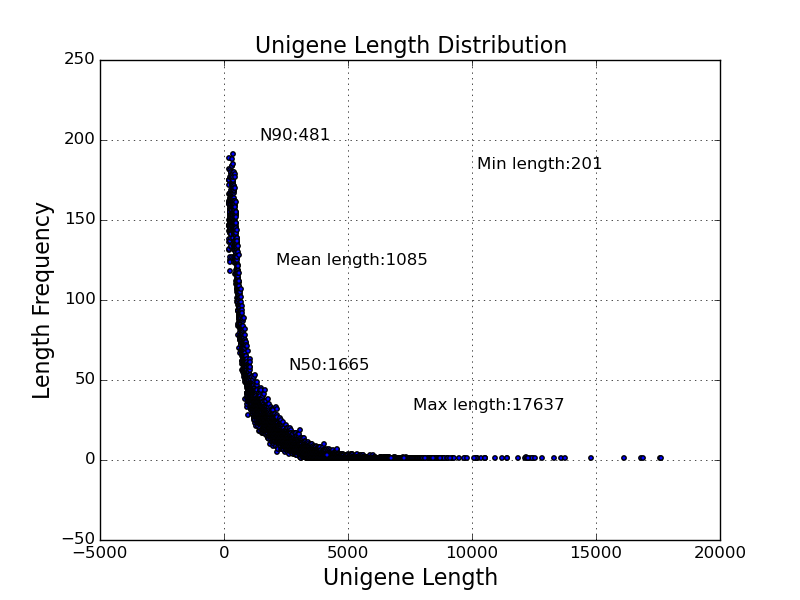

Supplement: Supplementary file 2 — The distribution of unigene size. (PNG 51 kb) [file 12985_2017_821_MOESM2_ESM.png]
